# Posterior allograft sacroiliac joint fusion with pre-operative mapping

**DOI:** 10.1016/j.inpm.2025.100581

**Published:** 2025-04-04

**Authors:** Chris Bovinet, Robert Moghim, Max Y. Jin, Alaa Abd-Elsayed

**Affiliations:** a The Spine Center of SE Georgia, Brunswick, GA, USA; b Colorado Pain Care, Denver, CO, USA; c Department of Anesthesiology, University of Wisconsin School of Medicine and Public Health, Madison, WI, USA

**Keywords:** Sacroiliac joint fusion, Sacroiliac joint dysfunction, Pre-operative mapping, Chronic pain

## Abstract

**Background:**

Low back pain is a highly prevalent and disabling condition. Sacroiliac joint dysfunction is prevalent in up to 62 % of some populations and is a common origin of low back pain. The posterior approach for minimally invasive sacroiliac joint fusion with an allograft is still relatively novel, with limited studies examining its safety and efficacy.

**Objective:**

The objective of our study was to analyze changes in pain and opioid usage for patients who underwent this procedure with pre-operative mapping after exhausting conservative treatment methods.

**Methods:**

This was a single-center, retrospective study with all cases completed by a single interventional pain physician. Outcomes regarding pain and opioid usage were extracted from electronic medical records, Georgia Prescription Drug Monitoring Program reports, and all other available state databases for 208 consecutive patients who underwent the minimally invasive sacroiliac joint fusion procedure with the LinQ Fusion Implant (PainTeq, Tampa, FL) after pre-operative mapping between August 2019 and October 2022. Pain was assessed using the Numerical Rating Scale (NRS), and opioid consumption was measured using Morphine Milligram Equivalents (MME).

**Results:**

NRS scores decreased from 7.23 ± 1.82 at baseline to 1.16 ± 1.35 at the final available follow-up (p < 0.001). Pain improvements ranged from 40 to 100 %, and all patients reported at least some improvement post-intervention. 205 of the 208 patients reported an improvement in pain of at least 50 %. MME reduced from 20.74 ± 26.33 mg to 10.00 ± 18.69 mg (p < 0.001).

**Conclusion:**

Posterior allograft sacroiliac joint fusion significantly reduces pain and opioid consumption. Pre-operative mapping is beneficial but requires more evidence to elucidate its role in optimizing implant placement.

## 1 Introduction

Low back pain (LBP) is the most common cause of disability in the world and the fifth leading reason for seeking a physician in the United States [1,2]. In addition to impacting physical function, LBP reduces mental health and increases financial burden [3]. Despite the repercussions, LBP is often under-researched compared to conditions like cardiovascular disease and cancer because it is seldom fatal [4].

The sacroiliac joint (SIJ) is a common origin of the LBP [5]. The SIJ is the part of the pelvis where the sacrum joins the ilium on each side [6]. It is a synovial joint that allows for specific rotational and translational movements but is otherwise immobile. The joint transfers load from the trunk to the pelvis and lower extremities [5]. SIJ dysfunction occurs when the joint has altered mobility and causes pain. Several events, including pregnancy, trauma, gait abnormalities, leg length discrepancy, spinal stenosis, and repetitive stress, can cause SIJ dysfunction [5,7]. This dysfunction is estimated to be prevalent in up to 62 % of some populations [8]. In addition to LBP, SIJ dysfunction can cause pain in the thighs, legs, groin, and buttocks.

Conservative treatments for SIJ dysfunction include physical therapy, nonsteroidal anti-inflammatory drugs, intra-articular injections, and radiofrequency ablation [9]. SIJ fusion is a surgical treatment for SIJ dysfunction that is reserved for when conservative management strategies fail, and pain persists for six or more months. The procedure was first described with an open approach in the early 1900s. Open SIJ fusion was hindered by long surgical times, extensive recovery, and high complication rates. Today, minimally invasive SIJ fusion is the most common, while the open approach is reserved for patients with traumatic fractures [10].

The posterior approach for minimally invasive SIJ fusion with an allograft is still relatively novel, with limited studies examining its safety and efficacy [[10], [11], [12]]. While this procedure has been performed in the past without joint mapping, we question whether pre-operative mapping can improve outcomes due to the common occurrence of varying SIJ anatomy. We present a retrospective study on patients with SIJ dysfunction who underwent this procedure at a single center. We aim to assess changes in pain and opioid consumption following posterior allograft SIJ fusion with pre-operative mapping.

## 2 Methods

This single-center, retrospective study received an IRB exemption from the WCG IRB. A single interventional pain physician completed all cases.

### 2.1 Patient selection and data collection

Electronic medical records were collected for all 208 consecutive patients who underwent the minimally invasive SIJ fusion procedure with the LinQ Fusion Implant (PainTeq, Tampa, FL) between August 2019 and October 2022. Inclusion criteria consisted of SIJ dysfunction diagnosis and failure of conservative therapies. Two successful diagnostic blocks (>75 % improvement in pain) were also required for inclusion. All diagnostic injections were contrast-enhanced with 0.5 cc of contrast or less and 1.5–2 cc of 0.5 % Bupivacaine. No steroids were injected in diagnostic blocks. Patients were excluded if they did not report information regarding our outcomes of interest: changes in pain and opioid consumption (i.e., missing data at baseline or follow-up). The Numeric Rating Scale (NRS) was used for the evaluation of pain, while opioid consumption was measured with Morphine Milligram Equivalents (MME). The NRS is an 11-point scale that allows patients to rate their pain from 0 to 10, with higher numbers indicating greater pain intensities. We defined Minimally Clinical Important Change (MCIC) as a 50 % or more remarkable improvement in pain. MME calculates the total opioid consumption across multiple drugs taken by patients using morphine as the standard measurement unit. The reported MME was further confirmed with the Georgia Prescription Drug Monitoring Program (PDMP) and all other available state databases. Follow-up NRS scores were extracted for patients post-operative, six weeks, six months, and the final available follow-up. MME scores were extracted at the final available follow-up. Statistical analysis was completed with a paired T-test using the SPSS 22 software. A p-value ≤0.05 indicated statistical significance.

### 2.2 Pre-operative mapping

**Fig. 1:**
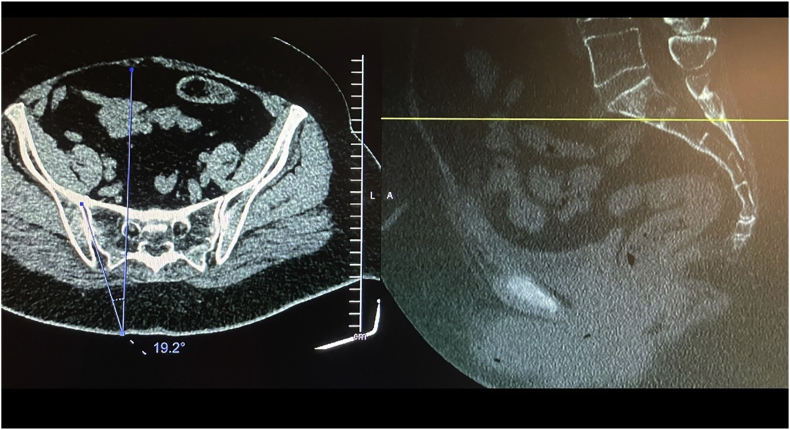
Contralateral oblique angle measurement. This figure depicts how the contralateral oblique angle was measured on CT pre-operatively.

**Fig. 2:**
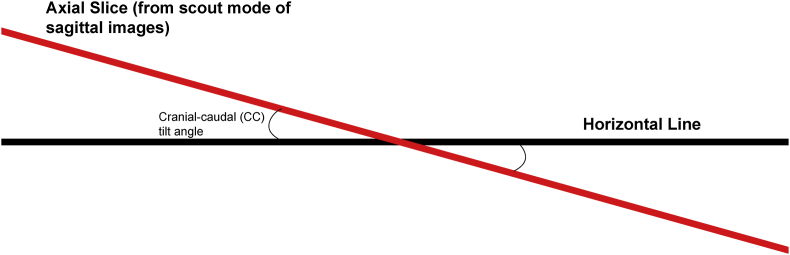
Cranial-Caudal tilt diagram. This figure depicts how the angle for the Cranial-Caudal tilt was measured.

**Fig. 3:**
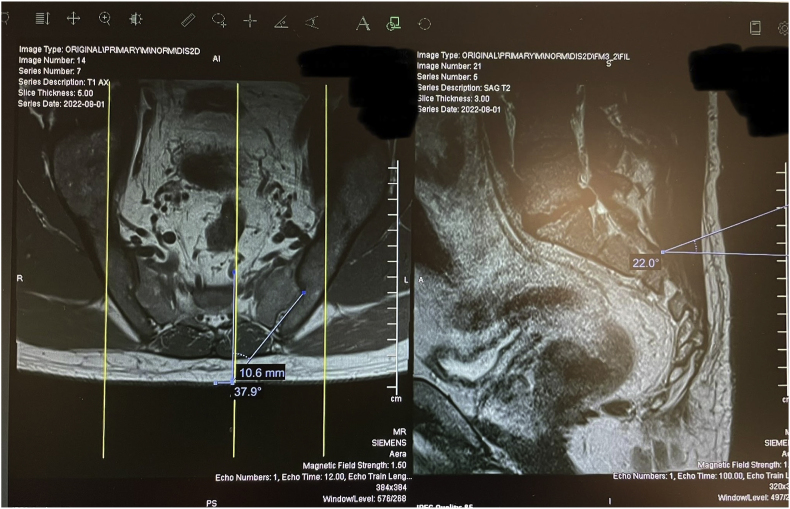
Axial and Mid-sagittal MRI images of SIJ with bilateral varus angulations. This figure depicts an example where the contralateral oblique angle is 37.9° (left image) and Cranial-Caudal tilt angle is 22° (right image).

Before the procedure, all patients underwent imaging studies to map their SIJ anatomies pre-operatively. An MRI or CT scan was taken of the patient's sacral/pelvic region. Upon review of these images, the best straight path into the posterior SIJ, located near the S1 foramen, was identified. Specific attention in axial slices was directed towards hardware that may impede the path of the tools as well as variations of normal anatomy, including varus or valgus angulations, S-shaped curvatures, and areas of the joint where the width of the joint was too wide to achieve distraction and bicortical contact with an 8 mm wide allograft implant. Examples of what may be encountered with a posterior approach include the interosseous and posterior SI ligaments and median and lateral sacral arteries [13]. This approach spares the gluteus maximus muscle and mitigates the risk of sacral nerve root injury, superior gluteal artery, and nerve root injury that can occur with the lateral approach. The contralateral oblique angle of the C-arm was determined as the angle measured between the straight line into the SIJ being treated and the line perpendicular to the sacrum in an axial view (adjusting for any rotation of the sacrum) (Fig. 1). The C-arm's cranial-caudal (CC) tilt was measured following this determination. The angle of the axial slice produced by the scout mode on the sagittal images was compared to a horizontal line. If the axial slice is perfectly horizontal, then no CC tilt of the C-arm is required. If not wholly horizontal, tilt the C-arm by the angle formed between the axial slice (scout line) and the horizontal line (Fig. 2). For example, if the slice is 22° from horizontal, that is your CC tilt angle of your C-arm (Fig. 3). This may change some as the sacrum's nutation (or flexion) can change from supine for the CT images to prone for the procedure. When viewed in lateral fluoroscopy (with iliopectineal lines appearing as one), the needle inside the joint would seem like the scout line on your sagittal images from your CT (Fig. 4a/4b). Lastly, the width (medial-lateral length) of the joint space was measured to verify that it was 6 mm or less for our 8 mm allograft. This ensures bicortical contact of the allograft after decortication of the cartilaginous surface into the cortical bone with the mechanical decortication device to effectively prepare the joint for fusion. If patients underwent bilateral SIJ fusion, both joints were mapped separately to account for anatomical differences.

**Fig. 4a:**
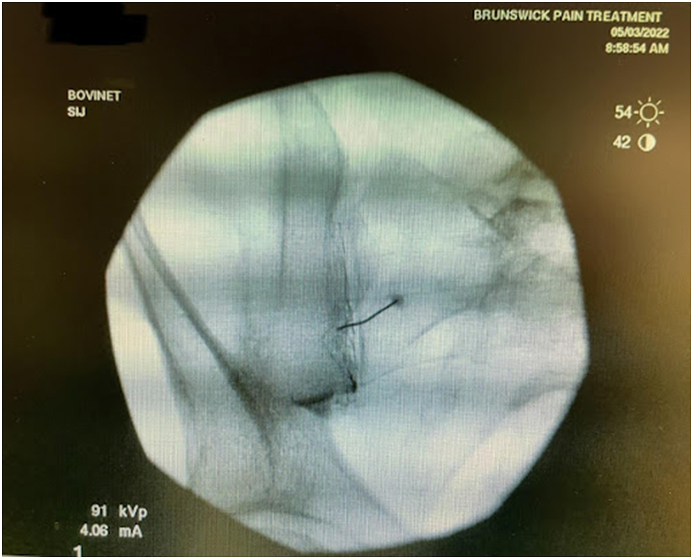
Contralateral oblique fluoroscopic image of needle trajectory. This figure depicts the needle from a diagnostic block and how the trajectory should be the same as the scout line extending across the joint on sagittal images of MRI or CT.

**Fig. 4b:**
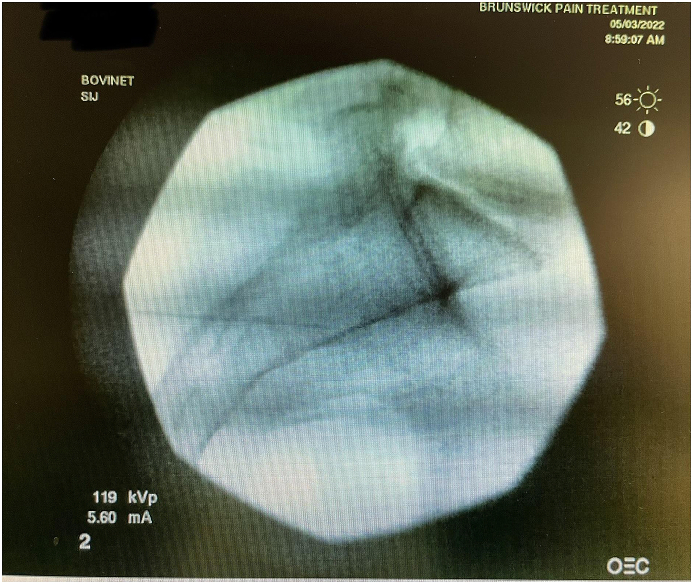
Lateral fluoroscopic image of needle trajectory. This figure depicts the needle from a diagnostic block and how the trajectory should be the same as the scout line extending across the joint on sagittal images of MRI or CT.

### 2.3 Surgical technique

**Fig. 5:**
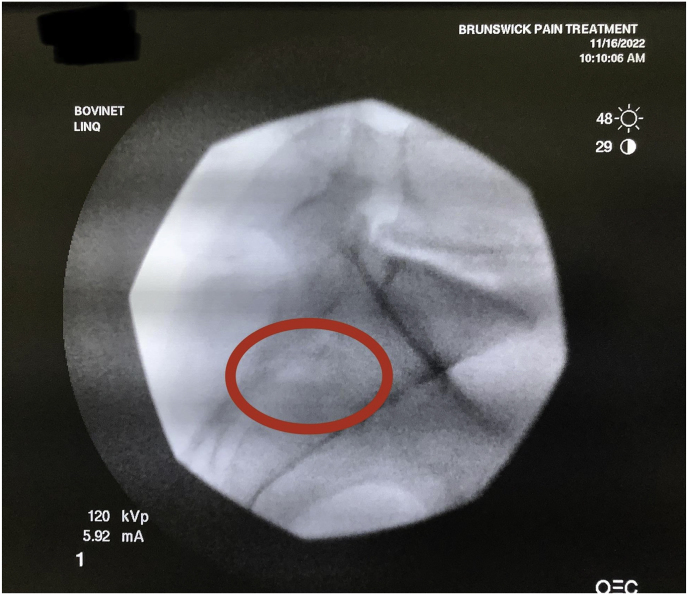
Final intraoperative lateral image. This figure depicts the final lateral fluoroscopic image that confirms successful implant placement.

**Fig. 6:**
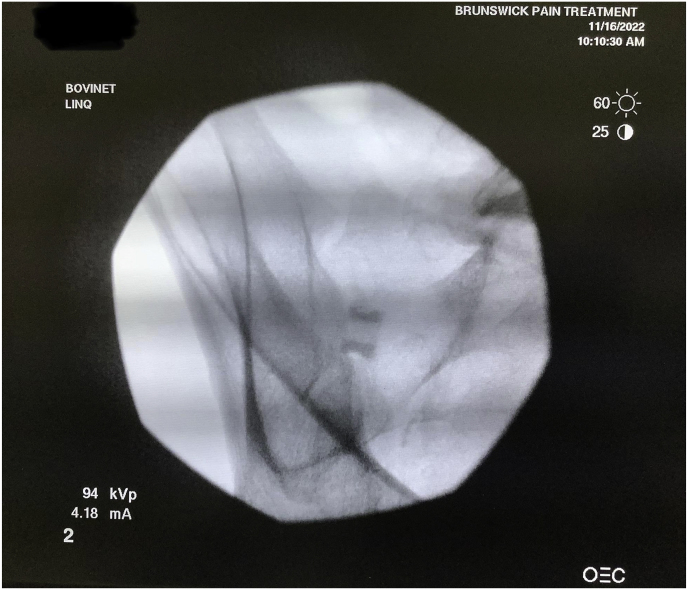
Final intraoperative contralateral oblique image. This figure depicts the final contralateral oblique fluoroscopic image that confirms successful implant placement.

Following informed consent, the patient is placed under moderate intravenous sedation after being placed in a prone position. The patient is draped, leaving the low back and upper buttocks exposed for access. Next, it is verified that the patient is lying flat for an accurate AP view at 0° tilt before tilting contralaterally to the measured angle. A single AP fluoroscopic image is obtained to ensure the image is AP and no airplane of the table is needed. Once the actual AP is confirmed, the fluoroscope is rotated contralateral oblique to the measured angle of the joint from the axial slice, and the CC tilt is calculated from the sagittal image. The obliquity measured on pre-operative CT was the exact fluoroscopic contralateral oblique angle utilized. Then, a 5-inch 22-gauge spinal needle is inserted into the skin and into the SIJ to be treated. Appropriate needle placement is confirmed in contralateral oblique and lateral fluoroscopic images. Local anesthesia is provided in 1-inch vertical lines around the needle and then through the needle into the joint and along the surgical tract as the needle is withdrawn. A 1-inch incision is made in the skin overlying the target area, and a Steinmann pin is inserted into the joint space without drilling. Appropriate Steinmann pin placement is confirmed with contralateral oblique and lateral fluoroscopic images. Next, separation in the joint is obtained by inserting a tissue dilator and working a cannula. The Steinmann pin and tissue dilator are removed, and the joint space is decorticated with a broach and rasp. The decorticator is removed by unscrewing the handle from the base and using a reverse slap hammer. The allograft implant is placed using an inserter after the large graft window is packed with demineralized bone matrix (DBM). Fluoroscopic images confirm successful implant placement with final lateral (Fig. 5) and contralateral oblique (Fig. 6). Deep palpation is applied to the implant to ensure anatomic placement before irrigating and closing the wound. Following the procedure, the patient is discharged home on the same day.

## 3 Results

### 3.1 Patient demographics

Our study included 208 consecutive patients, 130 females and 78 males (Table 1). All patients were diagnosed with SIJ dysfunction. The mean age was 70.42 ± 10.64 years. Seventy-one patients underwent left SIJ fusion, 70 underwent right, and 67 underwent bilateral. The mean final available follow-up length for data collection was 18.02 ± 14.92 months.

**Table 1:** Patient demographics.

**Total Patients**	208
**Gender**	Female – 130
	Male – 78
**Race**	Caucasian – 175
	African American – 32
	Asian – 1
**SIJ Fusion Laterality**	Left – 71
	Right – 70
	Bilateral – 67
**Mean Age (SD)**	70.42 years (10.64)
**Mean BMI (SD)**	29.82 kg/m^2^ (6.15)
**Mean Final Available Follow-up Time (SD)**	18.02 months (14.92)

### 3.2 Patient outcomes

All 208 patients recorded NRS and opioid usage. Pain scores decreased from a mean of 7.23 ± 1.82 pre-operatively to 0.95 ± 1.55 post-operatively (p < 0.001) and 1.16 ± 1.35 at the final available follow-up (p < 0.001) (Table 2). The mean final available follow-up time was 18.02 months ±14.92. Improvements in pain ranged from 40 % to 100 %, with all patients reporting at least some pain relief post-intervention. 205 out of 208 patients met the MCIC of 50 % or more improvement in pain. NRS scores decreased from 7.22 ± 1.82 to 2.72 ± 2.47 at six weeks (n = 201, p < 0.001) and 7.31 ± 1.69 to 1.00 ± 1.19 at six months (n = 153, p < 0.001) (Table 2). Patients without fixed follow-up data were deceased (unrelated to the procedure) (n = 2), or data was unavailable. For opioid usage, the mean MME consumption decreased from 20.74 ± 26.33 mg at baseline to 10.00 ± 18.59 mg at the final available follow-up. The average 84 % reduction in pain levels and 51.8 % decrease in opioid consumption at the final available follow-up were both statistically significant (p < 0.001). No complications were reported.

**Table 2:** Changes in NRS scores.

	Mean NRS score (SD)	p-value	Sample Size
Baseline	7.23 (1.82)	p < 0.001	208 patients
Post-operative (10–14 days)	0.95 (1.55)		
Baseline	7.22 (1.82)	p < 0.001	201 patients
6 week follow-up	2.72 (2.47)		
Baseline	7.31 (1.69)	p < 0.001	153 patients
6 month follow-up	1.00 (1.19)		
Baseline	7.23 (1.82)	p < 0.001	208 patients
Final available follow-up	1.16 (1.35)		

## 4 Discussion

Using an allograft, we present the first study utilizing pre-operative mapping for posterior SIJ fusion. We demonstrated that patients who were diagnosed with SIJ dysfunction experienced significant reductions in pain (84 % improvement) and opioid consumption (51.8 % reduction) after the allograft implant. Our results indicate greater pain outcomes than previously reported in the literature. Two high-powered studies, including a comparison cohort, have examined the efficacy of minimally invasive SIJ fusion using a lateral approach [14,15]. Whang et al. in the INSITE study, reported an average 63.9 % reduction in pain (82.3–29.8 [100 mm VAS]) at six months for patients who underwent SIJ fusion as compared to a 14.7 % reduction (82.2–70.4 [100 mm VAS]) in non-surgical management patients [14]. While MME was not measured in this study, the percentage of patients taking opioids decreased from 67.6 % to 58 % in SIJ fusion patients and increased from 63 % to 70.5 % in non-surgical management patients. There were 133 reported adverse events in patients who underwent lateral SIJ fusion, with two that were device-related and 17 related to the procedure. Dengler et al. in the iMIA study, reported a 45.3 average reduction in 100 mm VAS pain scores from 77.7 at baseline for the SIJ fusion group (58.3 % improvement) and an average 11.3 point reduction from 73.0 at baseline in the conservative management group [15]. The percentage of patients taking opioids in the SIJ fusion group decreased from 56 % to 33 %. In contrast, the percentage taking opioids in the conservative management group decreased from 47.1 % of patients to 45.7 %. Thirty-nine adverse events were reported in the lateral SIJ fusion group, with four related to the device or procedure.

Our present study has also demonstrated greater pain improvements than previously published reports using the same SIJ fusion implant without pre-operative mapping. Sayed et al. published the first study examining the efficacy of the LinQ Fusion Implant. They reported that patients experienced average pain reductions of 71.9 % (7.74–3.75 [NRS]) and 59.6 % (6.32–2.43 [NRS]) in patients with or without a history of previous lumbar surgeries, respectively (mean follow-up = 612.2 days; SD = 116.1) [11]. Changes in opioid consumption was not assessed and one device-related adverse event was reported. Calodney et al. reported results from the SECURE study where patients reported an average of 46.8 % improvement in pain (74.6–39.7 [100 mm VAS]) six months after being treated with the posterior allograft implant [10]. Data regarding opioid consumption was not reported. Three adverse events occurred, with none related to the device. Most recently, Moghim et al. published results demonstrating a 68.9 % improvement in pain (8.71–2.71 [10 cm VAS]) at 12 months post-SIJ fusion with the LinQ Fusion Implant [12]. MME reduced from 85.95 to 66.29 and no adverse events were reported.

Due to the complex anatomy of the SIJ, the use of intraoperative fluoroscopy may not be sufficient for finding an optimal view of the target. Furthermore, fluoroscopy can fall short of identifying SIJ anatomical variations that alter the procedure's approach [16]. SIJ anatomical variations are common, with most studies reporting a prevalence ranging from 25.7 to 35.7 % [[17], [18], [19]]. Some studies have even reported a prevalence of 52.2 % and 82.8 % in their study cohorts [20,21]. Prassopoulos et al. were the first to describe the different types of anatomical variations [18]. The variations include accessory joint, iliosacral complex, bipartite iliac bony plate, crescent bony plate, semicircular defects, and secondary ossification centers. Specific demographic groups that are more likely to exhibit anatomical variations include women, elderly adults, obese adults, multiparous women, and adults with axial spondyloarthritis or other forms of mechanical SIJ disease [22,23]. Pre-operative mapping with CT or MRI is essential to the minimally invasive SIJ fusion set-up because it allows the clinician to prepare for any SIJ anatomical variations.

**Fig. 7:**
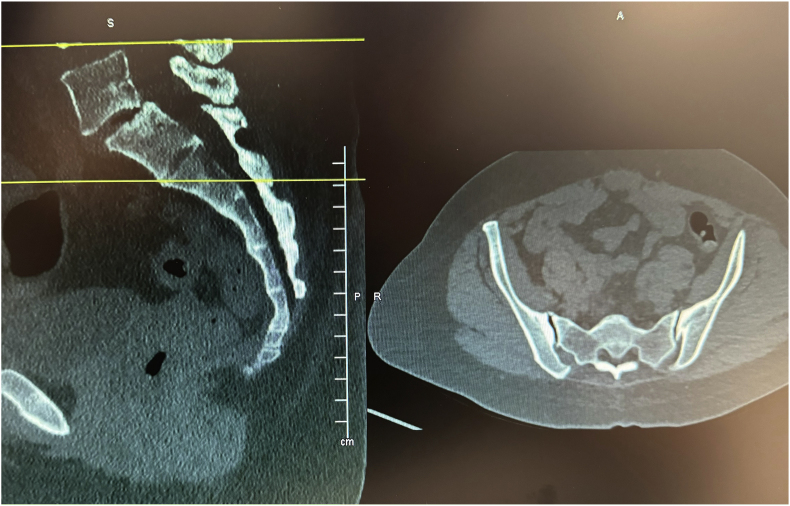
Accessory SIJ and varus angulation with S-curvature. This figure depicts a CT image where accessory SIJ and varus angulation with S-curvature were identified during pre-operative mapping.

**Fig. 8:**
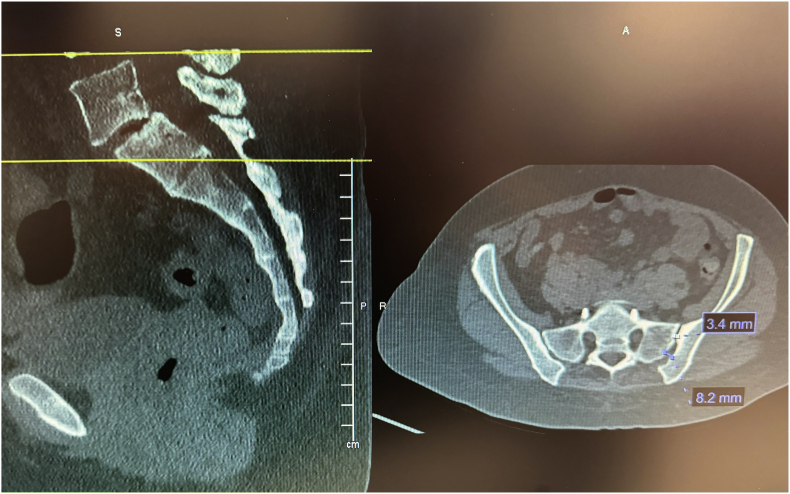
SIJ space width measurements. This figure depicts the measurement of SIJ space width where the posterior recess space was 8.2 mm, which is too broad for the implant, leading to our target location in the actual articulatory portion of the joint, which was 3.4 mm in width.

**Fig. 9:**
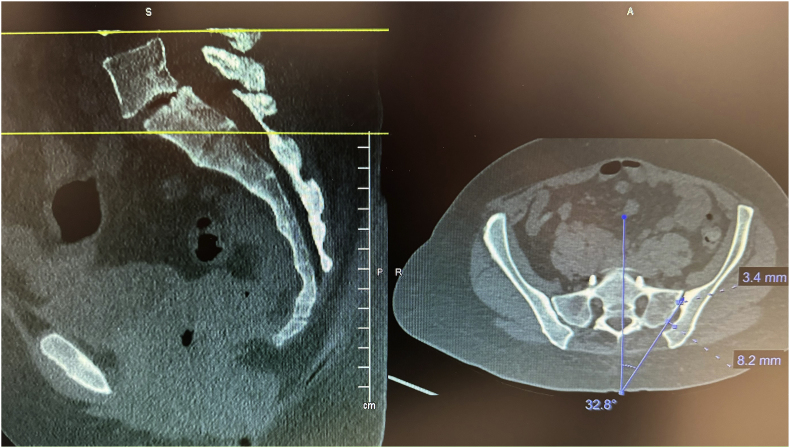
Contralateral oblique angle measurement. This figure depicts the measurement of the contralateral oblique angle for the C-arm as the angle between the straight line into the SIJ being treated and line perpendicular to the sacrum in an axial view.

**Fig. 10:**
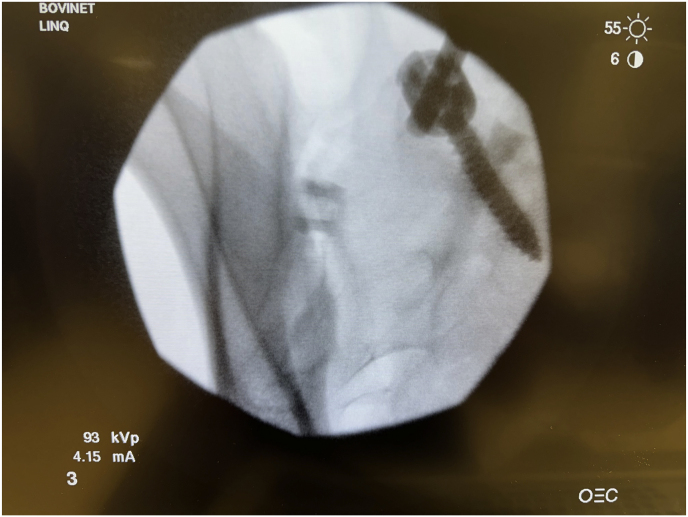
Final lateral view. This figure depicts the final lateral fluoroscopic image confirming the successful placement of the implant.

An example from our study was during a case where a patient was identified to have accessory SIJ and varus angulation with S-curvature during pre-operative mapping (Fig. 7). After measurement of the joint space, it was determined that the posterior recess would be too wide to accommodate the allograft implant. The actual articulatory portion of the joint was then identified (Fig. 8). The contralateral oblique angle for the C-arm was quickly identified when measuring the trajectory into the target space and the line perpendicular to the sacrum in an axial view (Fig. 9). Using the contralateral oblique angle measured on pre-operative CT, the position of the spinal needle and the Steinman pin were checked to ensure they appeared precisely as the scout line appeared on a mid-sagittal image. After confirmation, the implant was advanced to the anterior body of the sacrum, and the patient experienced a successful implant (Fig. 10). Pre-operative mapping can also reduce fluoroscopy time and intra-operative time. Our study found fluoroscopy time consistently 0.5 min compared to the 1.3 min average in the SECURE study [10]. While the impact of anatomical variations has not been reported for minimally invasive SIJ fusion with a posterior approach, Wessell et al. examined the effect with a lateral approach [23]. Their study found that, compared to patients with normal anatomy, patients with accessory joint or bipartite iliac bony plates experienced more remarkable outcomes. In contrast, patients with semicircular defects or crescent iliac bony plates experienced diminished outcomes.

Posterior and lateral SIJ fusion are the current minimally invasive approaches with published data. Lateral SIJ fusion was the first minimally invasive approach introduced and has received the most research attention. Compared to lateral, posterior SIJ fusion may present an even less invasive approach as it requires less surgical dissection, leading to lower operative times and quicker recovery [9]. There are also no weight-bearing restrictions after posterior allograft SIJ fusion compared to six weeks of restrictions with the lateral approach surgery. However, the lack of published literature has led the International Society for the Advancement of Spine Surgery (ISASS) not to recommend the dorsal approach in the most recent policy update in 2020. Our present study adds to the clinical evidence available and hopes to change the ISASS's recommendations concerning posterior SIJ fusion in future policy updates.

Our study contains some limitations that need to be acknowledged. The retrospective study design presents a risk for reporting bias and incomplete data. Additionally, we could not obtain data regarding changes in function or quality of life, which can be impacted by SIJ dysfunction. Lastly, post-operative CT scans were not available for all patients. Future studies are warranted to assess changes in pain, opioid usage, function, and quality of life with a prospective study design. These studies should also include a large sample size with a comparative or sham group.

## 5 Conclusion

Our study demonstrated that posterior SIJ fusion using an allograft implant significantly reduces pain and opioid consumption in patients with SIJ dysfunction. Implementing pre-operative mapping exposes any anatomical variations and minimizes peri-operative radiation exposure. Whether pre-operative mapping can optimize implant placement and improve outcomes requires further investigation with comparative study designs.

## Informed consent statement

Patient consent was not required as this was a retrospective chart review with no contact with the patients.

## Author contributions

Conceptualization: C.B. and A.A-E; Data Acquisition: C.B.; Data analysis and interpretation: C.B., R.M., M.Y.J., and A.A-E; writing—original draft preparation: C.B. and M.Y.J.; writing—review and editing: C.B., R.M., M.Y.J., and A.A-E. All authors have read and agreed to the published version of the manuscript.

## Institutional review board statement

The study received an IRB exemption from the WCG IRB.

## Data availability statement

Data is available upon request to the corresponding author.

## Funding

This study was supported by a research grant from PainTeq (Tampa, FL, USA). The funders had no role in the study's design, in the collection, analyses, or interpretation of data, in the writing of the manuscript, or in the decision to publish the results.

## Declaration of competing interest

The authors declare the following financial interests/personal relationships which may be considered as potential competing interests:Chris Bovinet reports a relationship with PainTeq that includes: consulting or advisory. Chris Bovinet reports a relationship with Nevro that includes: consulting or advisory. Chris Bovinet reports a relationship with Boston Scientific that includes: consulting or advisory. Chris Bovinet reports a relationship with Spinal Simplicity that includes: consulting or advisory. Chris Bovinet reports a relationship with Vertos that includes: consulting or advisory. Robert Moghim reports a relationship with Abbott that includes: consulting or advisory. Robert Moghim reports a relationship with PainTeq that includes: consulting or advisory. Robert Moghim reports a relationship with Vertos that includes: consulting or advisory. Robert Moghim reports a relationship with Curonix that includes: consulting or advisory. Alaa Abd-Elsayed reports a relationship with Medtronic that includes: consulting or advisory. Alaa Abd-Elsayed reports a relationship with Curonix that includes: consulting or advisory. Alaa Abd-Elsayed reports a relationship with Avanos that includes: consulting or advisory. If there are other authors, they declare that they have no known competing financial interests or personal relationships that could have appeared to influence the work reported in this paper.
